# The Donnan equilibrium is still valid in high-volume HDF

**DOI:** 10.1177/03913988241296699

**Published:** 2024-11-13

**Authors:** Malte Gross, Alfred Gagel, Andreas Maierhofer

**Affiliations:** 1 Faculty of Mechatronics and Medical Technology, Ulm University of Applied Sciences, Ulm, Baden-Württemberg, Germany; 2 Global Systems Engineering, VS Machines, Care Enablement, Fresenius Medical Care Deutschland GmbH, Schweinfurt, Bavaria, Germany

**Keywords:** Hemodialysis, hemodiafiltration, Donnan effect, solute kinetics in dialysis, Nernst-Planck equation, sodium balance, hemofiltration

## Abstract

Clinical studies have shown that hemodiafiltration reduces morbidity and mortality of dialysis patients compared to hemodialysis alone. This is attributed to its superior middle molecule clearance compared to standard hemodialysis. However, doubts arose as to whether a high convective flux through the dialyzer membrane has an influence on the equilibrium concentration of small ions, especially that of sodium. Due to the presence of negatively charged impermeable proteins on the blood side, the Gibbs-Donnan effect leads to an asymmetric distribution of membrane permeable ions on both sides of the membrane. In thermodynamic equilibrium, the concentrations of those ions can easily be calculated. However, the convective fluid flow leads to deviations from thermodynamic equilibrium. In this article, the effect of a convective flow on the ion distribution across a semipermeable membrane is analyzed in a theoretical model. Starting from the extended Nernst-Planck equation, including diffusive, convective, and electrostatic effects, a set of differential equations is derived. An approximate solution for flow speeds up to 0.1 ms^−1^ as well as a numerical solution are given. The results show that in any practical dialysis setting the convective flow has negligible influence on the electrolyte concentrations.

## Introduction

**Figure 1.**
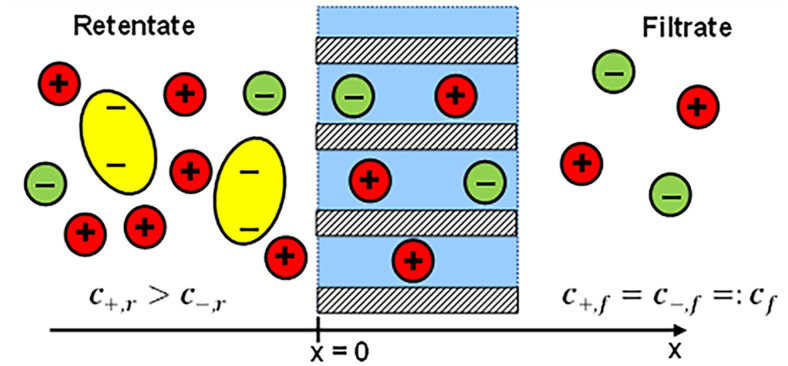
Schematic view of the ion distribution on both sides on both sides of a filtration membrane. On the left (retentate) side of the membrane negatively charged protein molecules (yellow) are present which are too large to pass through the membrane pores. Small monovalent ions can pass and are present on both sides. The one-dimensional coordinate *x* is defined as the distance from the left membrane boundary, the origin has been set at the left boundary of the membrane.

Blood purification by hemodialysis is based upon solute transport through a semi-permeable microporous membrane (Figure 1). Small molecules pass through it, while particles larger than the membrane pores are retained. Generally, the driving forces of transport through a membrane are gradients in concentration and pressure. Additionally, charged solutes, like electrolytes or charged proteins, are subject to electrostatic forces.

These driving forces are the origin of the two basic mechanisms of solute removal through dialysis: diffusion and convection.

According to Fick’s law, solute transport by diffusion depends on the concentration gradient of the solutes and on their particle size. The convective molar flux $J_{a}$ of a solute is equal to the product of the molar solute concentration $c_{r}$ on the retentate side and the total filtration volume flow $Q_{f}$ ^1,2^:

(1) $$J_{a}=(1-\sigma )Q_{f}c_{r}.$$

σ is the Staverman reflection coefficient, which increases with increasing molecular weight.

An ideal membrane has a sharp cut-off threshold for solutes. Ideally, σ would be equal to 0 for all permeable molecules and increase quickly to 1 for the larger molecules to be retained.^3^ For small ions like Na^+^ and Cl^−^, the reflection is negligible whereas large proteins like albumin cannot penetrate the membrane so that their reflection coefficient is nearly 1.^4^

Charged particles are also subject to electrostatic interaction. One consequence of this is the Gibbs-Donnan effect.^5^ Large molecules carrying negative charges like albumin are only present on the retentate side. Charge neutrality both in the retentate as well as in the filtrate implies that the concentrations of a permeable ion species on both sides of the membrane are different. In the simple case of only two membrane-permeable ions with opposite and equal charge, the concentrations in retentate and filtrate in the Gibbs-Donnan equilibrium are given by^6^:

(2) $$c_{+,r}c_{-,r}=c_{f}^{2}$$

where $c_{+,r}$ and $c_{-,r}$ are the retentate concentration of positive and negative permeable ions and $c_{f}=c_{+,f}=c_{-,f}$ their filtrate concentration, respectively.

The Gibbs-Donnan ratio $r_{d}$ is defined as the ratio of filtrate and retentate concentrations for each electrolyte:

(3) $$r_{d}:=\frac{c_{f}}{c_{+,r}}=\frac{c_{-,r}}{c_{f}}$$

In the case of no convective flow, equation (2) can be derived without explicitly assuming the system to be in thermodynamic equilibrium.^6^ High convective ultrafiltration flow rates lead to increased protein concentrations on the retentate side of the membrane. In the medical literature, contradictory opinions exist whether this has a relevant influence on the ionic concentrations in the filtrate. A theoretical analysis by Gotch^7^ claims a decreased net sodium removal, while the Donnan equilibrium relation (3) itself is still valid. Other authors claim a breakdown of the equilibrium induced by the high convective flow.^8^ Locatelli et al.^9^ propose to reduce the reinfusate sodium concentration by 8 mmol/L to avoid sodium accumulation. However, in-vivo studies did not find any change of fluxes of the permeable ions even at high transmembrane flows.^10,11^

In order to clarify this controversy, this article presents a theoretical model of ion transport based on first principles. The derivation starts with the extended Nernst-Planck-equation. For simplicity, the model comprises only three ionic solutes: A permeable cation (e.g. Na^+^), a permeable anion (e.g. Cl^−^) of opposite charge, and a charged non-permeable species (e.g. proteins like albumin or hemoglobin). The system is not explicitly assumed to be in thermodynamic equilibrium.

## Theoretical model of ion distribution

In order to keep the model manageable, some assumptions and simplifications are made:

1. The membrane is assumed flat and of infinitely large area. Its boundaries are parallel, the pores are cylindrical and their axes are perpendicular to the membrane surfaces. Therefore, all quantities depend only on a single coordinate x, which is the perpendicular distance from the retentate side boundary of the membrane (Figure 1).
2. Stationary conditions are assumed, all particle concentrations are time-independent.
3. The electrostatic forces acting on the ions are modeled by a bulk mean potential *U*(*x*) alone. Individual ion-ion interactions^5,6^ are neglected.
4. The membrane itself is not charged.

Three local driving forces act on an individual ion: Firstly, the drag produced by the transmembrane water flow, secondly, the concentration gradient giving rise to the diffusion and finally the electrostatic force due to the gradient of the electric potential *U*(*x*) created by the global charge distribution. All three contributions are included in the one-dimensional Nernst-Planck equation,^2,12,13^ extended by the convective flux term^14^ proportional to the flow speed $v_{f}\left(x\right)$ :

(4) $$\begin{array}{l} j_{i}(x)=-D_{i}\left(\frac{d}{dx}c_{i}\left(x\right)+\frac{z_{i}F}{RT}c_{i}\left(x\right)\frac{d}{dx}U(x)\right)\\ \;\;\;\;\;\;\;\;\;\;\;\;\;+v_{f}\left(x\right)c_{i}\left(x\right) \end{array}$$

$j_{i}\left(x\right)$ is the total flux, $D_{i}$ the diffusion coefficient, $z_{i}$ the valence, and $c_{i}\left(x\right)$ the molar concentration of ion species i at distance *x* > 0 from the left membrane surface, respectively. *F*, *R*, and *T* are Faraday constant, molar gas constant, and absolute temperature, respectively. Assuming a homogenous flow distribution in each cross section of the membrane pores and a constant pore diameter along the *x*-axis, the flow speed $v_{f}\left(x\right)$ does not depend on *x*. It can be expressed in terms of the total effective pore area *A* and the total transmembrane volume flow $Q_{f}$ :

(5) $$v_{f}=\frac{Q_{f}}{A}$$

Due to mass conservation, the total flux $j_{i}$ must satisfy the continuity equation:

(6) $$\frac{d}{dt}c_{i}\left(x\right)+\frac{d}{dx}j_{i}(x)=0$$

In the stationary case, the time derivative of the concentration vanishes and equation (6) simplifies to:

(7) $$\frac{d}{dx}j_{i}(x)=0$$

Thus, $j_{i}(x)=j_{i}$ is constant. Far away from the inner membrane boundary, the ion concentrations $c_{i}\left(x\right)\;$ and the electrostatic potential U(x) reach constant values *c_i_*,_ꝏ_ and U(∞), respectively. From (7) the constant total flux *j_i_* can be derived:

(8) $$j_{i}=v_{f}c_{i,\infty }$$

Introducing the Debye length *λ_D_* and the thermal voltage *U_T_*:

(9) $$\lambda_{D}=\sqrt{\frac{\varepsilon U_{T}}{F\sum_{i}z_{i}^{2}c_{i,\infty }}}\;\;\mathrm{and}\;\;\;U_{T}=\frac{RT}{F}$$

with the dielectric constant of water $\varepsilon =\varepsilon_{r}\;\varepsilon_{0}$ and the sum in the denominator running over all permeable ion species *i*, equation (4) can be rewritten:

(10) $$\begin{array}{l} \frac{v_{f}\lambda_{D}}{D_{i}}\left(1-\sigma_{i}\right)c_{i,\infty }=-\frac{d}{d\hat{x}}c_{i}\left(x\right)-z_{i}c_{i}\left(x\right)\frac{d}{d\hat{x}}\hat{U}(x)\\ \;\;\;\;\;\;\;\;\;\;\;\;\;\;\;\;\;\;\;\;\;\;\;\;\;\;\;\;\;\;\;\;\;\;\;\;\;\;\;+\frac{v_{f}\lambda_{D}}{D_{i}}c_{i}\left(x\right) \end{array}$$

In this equation, the dimensionless length $\hat{x}$ and potential $\hat{U}\left(\hat{x}\right)$ are defined as

(11) $$\hat{x}=\frac{x}{\lambda_{D}},\hat{U}\left(x\right)=\frac{U\left(x\right)}{U_{T}}.$$

The Debye length *λ_D_* is the characteristic length of the space-charge zone in the membrane pore. Only in this narrow zone diffusion occurs. In aqueous solutions, the lower bound of the ion strength is given by the autodissociation of water. Since $c_{i,\infty }\geq 10^{-7}\mathrm{mol}/L$ , *λ_D_* is in any case smaller than 98 nm. This is much less than the thickness of typical dialyzer membranes. Consequently, the membrane thickness has no significant influence on the diffusion process inside of the membrane pores.

Thus, the dimensionless factor $\frac{v_{f}\lambda_{D}}{D_{i}}$ in equation (10) is a characteristic property of the dynamics in the space-charge zone. It describes the ratio of convective and diffusive transport and can be interpreted here as the Peclet number of the space-charge region:

(12) $$Pe_{i}:=\frac{v_{f}\lambda_{D}}{D_{i}}.$$

Solving (10) for $\frac{d}{d\hat{x}}\hat{U}\left(x\right)$ and taking the derivative yields

(13) $$\begin{array}{l} z_{i}\frac{d^{2}}{d{\hat{x}}^{2}}\hat{U}\left(x\right)=\frac{{\left(\frac{d}{d\hat{x}}c_{i}\left(x\right)\right)}^{2}-c_{i}\left(x\right)\frac{d^{2}}{d{\hat{x}}^{2}}c_{i}\left(x\right)}{c_{i}{\left(x\right)}^{2}}\\ \;\;\;\;\;\;\;\;\;\;\;\;\;\;\;\;\;\;\;\;\;\;\;\;\;\;\;\;+Pe_{i}c_{i,\infty }\frac{\frac{d}{d\hat{x}}c_{i}\left(x\right)}{c_{i}{\left(x\right)}^{2}} \end{array}$$

The electrostatic potential *U*(*x*) depends on the individual ion concentrations *c_i_*. The relation between charge density ρ(x) and electrostatic potential is given by the Poisson equation:

(14) $$\Delta U\left(x\right)=-\frac{\rho \left(x\right)}{\varepsilon }$$

All individual ions with molar concentration $c_{i}$ and charge number $z_{i}$ contribute to ρ(x) :

(15) $$\rho \left(x\right)=F\underset{i}{\sum }z_{i}c_{i}\left(x\right)$$

In the one-dimensional case one obtains:

(16) $$\frac{d^{2}}{d{\hat{x}}^{2}}\hat{U}\left(x\right)=-\frac{1}{\underset{i}{\sum }z_{i}^{2}c_{i,\infty }}\underset{i}{\sum }z_{i}c_{i}\left(x\right)$$

In the case of two ions with equal but oppositely signed charges, for example Na^+^ and Cl^−^, or Mg^2+^ and SO_4_^2−^, charge neutrality in the filtrate far away from the charged layer implies

(17) $$c_{+,\infty }=c_{-,\infty }=c_{f}.$$

By normalizing the concentrations

(18) $$\frac{c_{i}\left(\lambda_{D}\hat{x}\right)}{c_{f}}=:\hat{c_{i}}\left(\hat{x}\right),i=+,-$$

and using the prerequisite $z_{+}=-z_{-}$ , equations (13) and (16) can be combined into two coupled nonlinear differential equations, one for each ion species:

(19) $$\begin{array}{c} \begin{array}{l} \frac{d^{2}}{d{\hat{x}}^{2}}{\hat{c}}_{+}\left(\hat{x}\right)=\frac{1}{2}\left({\hat{c}}_{+}{\left(\hat{x}\right)}^{2}-{\hat{c}}_{+}\left(\hat{x}\right){\hat{c}}_{-}\left(\hat{x}\right)\right)\\ \;\;\;\;\;\;\;\;\;\;\;\;\;\;\;\;\;\;\;\;\;\;\;\;+\frac{{\left(\frac{d}{d\hat{x}}{\hat{c}}_{+}\left(\hat{x}\right)\right)}^{2}}{{\hat{c}}_{+}\left(\hat{x}\right)}+Pe_{+}\frac{\frac{d}{d\hat{x}}{\hat{c}}_{+}\left(\hat{x}\right)}{{\hat{c}}_{+}\left(\hat{x}\right)} \end{array}\\ \begin{array}{l} \frac{d^{2}}{d{\hat{x}}^{2}}{\hat{c}}_{-}\left(\hat{x}\right)=\frac{1}{2}\left({\hat{c}}_{-}{\left(\hat{x}\right)}^{2}-{\hat{c}}_{+}\left(\hat{x}\right){\hat{c}}_{-}\left(\hat{x}\right)\right)\\ \;\;\;\;\;\;\;\;\;\;\;\;\;\;\;\;\;\;\;\;\;\;\;+\frac{{\left(\frac{d}{d\hat{x}}{\hat{c}}_{-}\left(\hat{x}\right)\right)}^{2}}{{\hat{c}}_{-}\left(\hat{x}\right)}+Pe_{-}\frac{\frac{d}{d\hat{x}}{\hat{c}}_{-}\left(\hat{x}\right)}{{\hat{c}}_{-}\left(\hat{x}\right)} \end{array} \end{array}$$

In its general form, this coupled nonlinear system of second-order differential equation cannot be solved analytically.

However, rearrangement of these equations allows do derive a simple expression for the filtrate concentration $c_{f}$ when the concentrations at the left membrane boundary ${\hat{c}}_{+}\left(0\right)$ and ${\hat{c}}_{-}\left(0\right)$ are given. Since the concentrations must be continuous, these are equal to the retentate concentrations ${\hat{c}}_{+,r}$ and ${\hat{c}}_{-,r}$ , respectively.

Dividing the first and second equation of (19) by ${\hat{c}}_{+}\left(\hat{x}\right)$ and ${\hat{c}}_{-}\left(\hat{x}\right)$ , respectively, and summing them yields:

(20) $$\begin{array}{l} \frac{d}{d\hat{x}}\left[\frac{\frac{d}{d\hat{x}}{\hat{c}}_{+}\left(\hat{x}\right)}{{\hat{c}}_{+}\left(\hat{x}\right)}+\frac{\frac{d}{d\hat{x}}{\hat{c}}_{-}\left(\hat{x}\right)}{{\hat{c}}_{-}\left(\hat{x}\right)}\right]=\\ -\frac{d}{d\hat{x}}\left[Pe_{+}\frac{1}{{\hat{c}}_{+}\left(\hat{x}\right)}+Pe_{-}\frac{1}{{\hat{c}}_{-}\left(\hat{x}\right)}\right] \end{array}$$

Integrating over $\hat{x}$ leads to:

(21) $$\begin{array}{l} \frac{\frac{d}{d\hat{x}}{\hat{c}}_{+}\left(\hat{x}\right)}{{\hat{c}}_{+}\left(\hat{x}\right)}+\frac{\frac{d}{d\hat{x}}{\hat{c}}_{-}\left(\hat{x}\right)}{{\hat{c}}_{-}\left(\hat{x}\right)}=\\ -Pe_{+}\frac{1}{{\hat{c}}_{+}\left(\hat{x}\right)}-Pe_{-}\frac{1}{{\hat{c}}_{-}\left(\hat{x}\right)}+K \end{array}$$

In the limit $\hat{x}\to \infty$ , the concentrations ${\hat{c}}_{+}$ and ${\hat{c}}_{-}$ both attain the constant value 1 and the derivatives become zero. This fixes the integration constant *K*:

(22) $$K=Pe_{+}+Pe_{-}$$

Multiplication with ${\hat{c}}_{+}\left(\hat{x}\right){\hat{c}}_{-}\left(\hat{x}\right)$ gives:

(23) $$\begin{array}{l} \frac{d}{d\hat{x}}\left({\hat{c}}_{+}\left(\hat{x}\right){\hat{c}}_{-}\left(\hat{x}\right)\right)=Pe_{+}\left({\hat{c}}_{+}\left(\hat{x}\right){\hat{c}}_{-}\left(\hat{x}\right)-{\hat{c}}_{-}\left(\hat{x}\right)\right)\\ \;\;\;\;\;\;\;\;\;\;\;\;\;\;\;\;\;\;\;\;\;\;\;\;\;\;\;\;\;\;\;\;\;\;\;\;\;\;+Pe_{-}\left({\hat{c}}_{+}\left(\hat{x}\right){\hat{c}}_{-}\left(\hat{x}\right)-{\hat{c}}_{+}\left(\hat{x}\right)\right) \end{array}$$

In the case of no convective flow, the Peclet numbers $Pe_{+}$ and $Pe_{-}$ are zero. Consequently, the product ${\hat{c}}_{+}\left(\hat{x}\right){\hat{c}}_{-}\left(\hat{x}\right)$ is constant in that case. Taking the limit $\hat{x}\to \infty$ shows that this constant is 1

(24) $${\hat{c}}_{+}^{\left(0\right)}\left(\hat{x}\right){\hat{c}}_{-}^{\left(0\right)}\left(\hat{x}\right)={\hat{c}}_{f}^{2}=1$$

The upper index (0) stands for the zero-convection case. Reverting the normalization (18) gives:

(25) $$c_{+}^{\left(0\right)}\left(\hat{x}\right)c_{-}^{\left(0\right)}\left(\hat{x}\right)={\left(c_{f}^{\left(0\right)}\right)}^{2}$$

Setting $\hat{x}=0$ proves the Gibbs-Donnan formula (3).

## Analytic and numerical solution of the model

In the non-convective case, an analytic solution of equation system (19) can be found:

(26) $$\begin{array}{c} {\hat{c}}_{+}^{\left(0\right)}\left(\hat{x}\right)={\left(\frac{1+\sqrt{r_{d}}-\left(1-\sqrt{r_{d}}\right)e^{-\hat{x}}}{1+\sqrt{r_{d}}+\left(1-\sqrt{r_{d}}\right)e^{-\hat{x}}}\right)}^{-2}\\ \begin{array}{c} {\hat{c}}_{-}^{\left(0\right)}\left(\hat{x}\right)={\left(\frac{1+\sqrt{r_{d}}-\left(1-\sqrt{r_{d}}\right)e^{-\hat{x}}}{1+\sqrt{r_{d}}+\left(1-\sqrt{r_{d}}\right)e^{-\hat{x}}}\right)}^{2}\\ =\;\;\;\;\;\;\frac{1}{{\hat{c}}_{+}^{\left(0\right)}\left(\hat{x}\right)} \end{array} \end{array}$$

where the Gibbs-Donnan-ratio $r_{d}$ (3) is given by.

(27) $$r_{d}={\hat{c}}_{-,r}^{\left(0\right)}=\frac{1}{{\hat{c}}_{+,r}^{\left(0\right)}}$$

In the presence of convective flow (26) is no longer valid. However, for small Peclet numbers $Pe_{+},Pe_{-}<<1$ , the influence of convection on the filtrate concentration $c_{f}\;$ can be approximated by perturbation theory. Under this assumption, the filtrate concentration without convection $c_{f}^{\left(0\right)}$ is equal to $\sqrt{c_{+,r}c_{-,r}}$ .

In the case of small convective flows, one expects the concentrations ${\hat{c}}_{+}\left(\hat{x}\right)$ and ${\hat{c}}_{-}\left(\hat{x}\right)$ to differ only slightly from the exact non-convective solutions ${\hat{c}}_{+}^{\left(0\right)}\left(\hat{x}\right)$ and ${\hat{c}}_{-}^{\left(0\right)}\left(\hat{x}\right)$ . In a first-order approximation, ${\hat{c}}_{+}\left(\hat{x}\right)$ and ${\hat{c}}_{-}\left(\hat{x}\right)$ on the right side of (23) can be replaced by ${\hat{c}}_{-}^{\left(0\right)}\left(\hat{x}\right)$ and ${\hat{c}}_{-}^{0}\left(\hat{x}\right)$ , respectively. Integration over $\hat{x}$ from 0 to ∞ gives the difference of the product ${\hat{c}}_{+}\left(\hat{x}\right){\hat{c}}_{-}\left(\hat{x}\right)$ between its filtrate and retentate values. Considering equation (18) yields:

(28) $$\begin{array}{l} 1-{\hat{c}}_{+,r}{\hat{c}}_{-,r}\\ =\overset{\infty }{\underset{0}{\int }}\left[Pe_{+}\left(1-{\hat{c}}_{-}^{\left(0\right)}\left(\hat{x}\right)\right)+Pe_{-}\left(1-{\hat{c}}_{+}^{\left(0\right)}\left(\hat{x}\right)\right)\right]d\hat{x} \end{array}$$

The integrals in equation (31) can be calculated analytically:

(29) $$\begin{array}{c} \overset{\infty }{\underset{0}{\int }}\left(1-{\hat{c}}_{+}^{\left(0\right)}\left(\hat{x}\right)\right)d\hat{x}={\left[4\frac{\left(\sqrt{r_{d}}-1\right)e^{-\hat{x}}}{\left(1-\sqrt{r_{d}}\right)e^{-\hat{x}}-1-\sqrt{r_{d}}}\right]}_{0}^{\infty }=2-\frac{2}{\sqrt{r_{d}}}\\ \overset{\infty }{\underset{0}{\int }}\left(1-{\hat{c}}_{-}^{\left(0\right)}\left(\hat{x}\right)\right)d\hat{x}={\left[4\frac{\left(\sqrt{r_{d}}-1\right)e^{-\hat{x}}}{\left(1-\sqrt{r_{d}}\right)e^{-\hat{x}}+1+\sqrt{r_{d}}}\right]}_{0}^{\infty }=2-2\sqrt{r_{d}} \end{array}$$

This results in:

(30) $$1-\frac{c_{+,r}c_{-,r}}{c_{f}^{2}}=2Pe_{+}\left(1-\sqrt{r_{d}}\right)+2Pe_{-}\left(1-\frac{1}{\sqrt{r_{d}}}\right)$$

Solving for $c_{f}$ and retaining only first-order terms in $Pe_{+}$ and $Pe_{-}$ gives a simple approximation for the deviation of the ion concentration in the filtrate $c_{f}$ from its Donnan equilibrium value $c_{f}^{\left(0\right)}$ :

(31) $$\begin{array}{c} \Delta c_{f}=c_{f}-c_{f}^{\left(0\right)}\\ \;\;\;\;\;\;\;\;\;\;\;=c_{f}^{\left(0\right)}\left[Pe_{+}\left(1-\sqrt{r_{d}}\right)+Pe_{-}\left(1-\frac{1}{\sqrt{r_{d}}}\right)\right] \end{array}$$

Using the definition of the Peclet numbers, this can be rewritten:

(32) $$\Delta c_{f}=c_{f}^{\left(0\right)}\lambda_{D}v_{f}\left(1-\sqrt{r_{d}}\right)\left(\frac{1}{D_{+}}-\frac{1}{D_{-}\sqrt{r_{d}}}\right)$$

In order to assess the validity of this approximative solution, equation set (26) was solved numerically using the bvp4c solver in the MATLAB package. Two second-order equations require four boundary conditions. Although $c_{+}\left(\hat{x}=0\right)$ and $c_{-}\left(\hat{x}=0\right)$ are given by the retentate concentrations $c_{+,r}$ and $c_{-,r}$ , respectively, $c_{f}$ is a priori unknown. Thus, the normalized concentrations ${\hat{c}}_{+}\left(\hat{x}=0\right)$ and ${\hat{c}}_{-}\left(\hat{x}=0\right)$ cannot be used as boundary values for the differential equations. However, the concentration ratio ${\hat{c}}_{+}\left(\hat{x}=0\right)/{\hat{c}}_{-}\left(\hat{x}=0\right)$ is independent of $c_{f}$ and was used as the first boundary condition. Since the numerical solver requires a finite right boundary ${\hat{x}}_{f}<\infty$ <∞ , ${\hat{x}}_{f}$ was set to ${\hat{x}}_{f}=100$ . At this point due to (17) the second boundary condition ${\hat{c}}_{+}\left({\hat{x}}_{f}\right){\hat{c}}_{-}\left({\hat{x}}_{f}\right)=1$ holds. Finally, at the right boundary, due to the absence of an electrostatic field, the ion concentrations converge to their constant limits. Thus, $\frac{d}{d\hat{x}}{\hat{c}}_{+}\left({\hat{x}}_{f}\right)=\frac{d}{d\hat{x}}{\hat{c}}_{-}\left({\hat{x}}_{f}\right)=0$ were chosen as the third and fourth boundary condition. A relative error tolerance of 10^–11^ was chosen and attained. Using right boundaries ${\hat{x}}_{f}$ of 90 and 110 yielded the same result within the error margin.

According to equation (18), the quotient between the retentate concentration $c_{+,r}$ and the numerical solution for ${\hat{c}}_{+}\left(\hat{x}=0\right)$ is the filtrate concentration $c_{f}$ . This allows to revert the normalization of the numerical results and yields the ion concentrations $c_{+}\left(x\right)$ and $c_{-}\left(x\right)$ .

## Results

As examples, three different salt solutions were analyzed under physiological conditions: NaCl, KCl, and NaHCO_3_. The reflection coefficients σ were assumed as zero for all permeable ion species. The diffusion constants at σ=25°C for potassium, sodium, chloride, and bicarbonate ions are $D_{K}^{\left(25\right)}=1.96\cdot 10^{-9}m^{2}s^{-1}$ , $D_{Na}^{\left(25\right)}=1.33\cdot 10^{-9}m^{2}s^{-1}$ , $D_{Cl}^{\left(25\right)}=2.03\cdot 10^{-9}m^{2}s^{-1}$ , and $D_{Bic}^{\left(25\right)}=1.19\cdot 10^{-9}m^{2}s^{-1}$ , respectively.^15^ At the typical physiological temperature of 37°C, the diffusivities can be calculated using the Stokes-Einstein rel-ation, and the water viscosity values, which are $\eta^{\left(25\right)}=0.89\mathrm{mPa}.s$ and $\eta^{\left(37\right)}=0.69\mathrm{mPa}.s$ .^16^ One obtains $D_{K}^{\left(37\right)}=2.53\cdot 10^{-9}m^{2}s^{-1}$ , $D_{Na}^{\left(37\right)}=1.72\cdot 10^{-9}m^{2}s^{-1}$ , $D_{Cl}^{\left(37\right)}=2.62\cdot 10^{-9}m^{2}s^{-1}$ , and $D_{Bic}^{\left(37\right)}=1.53\cdot 10^{-9}m^{2}s^{-1}$ .

**Figure 2.**
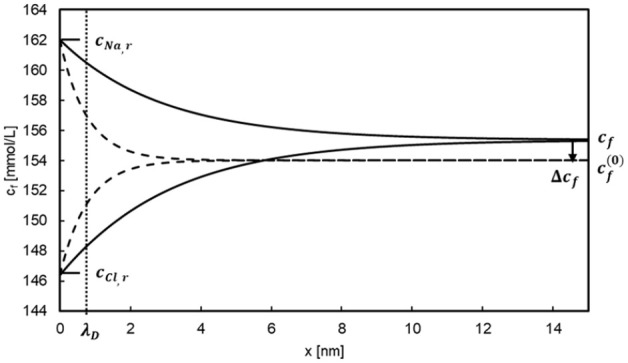
Na+ and Cl^−^ concentrations at zero transmembrane flow (dashed lines) and a flow speed of 10 ms^−1^ (solid lines). Retentate Na^+^ and Cl^−^ concentrations at *x* = 0 are 162 and 146.4 mmol/L, respectively, the temperature is 37°C. Gibbs-Donnan ratio is *rD* = 0.95. The Debye length is *λ*D = 0.77 nm.

Figure 2 depicts the anion and cation concentrations in the case of Na+ and Cl^−^ as permeable ions, calculated by numerically solving equation set (19) for $v_{f}=$ 0 and 10 ms^−1^, respectively. The concentrations are shown as a function of the distance from the left side of the membrane. The filtrate concentration in the zero-convection case was assumed as 154 mmol/L, the molarity of a 0.9% saline solution. The Gibbs-Donnan ratio was set to 0.95 which is a typical value for human plasma. In the zero-convection case equation (3) leads to retentate side Na^+^ and Cl^−^ concentrations of 162.0 and 146.4 mmol/L, respectively. Under these assumptions, the Debye length is 0.77 nm at a temperature of 37°C.

As expected, in the case of no convective flow, *c*_Na_(*x*) and *c*_Cl_(*x*) rapidly converge within a few nanometers to their equilibrium value of 154 mmol/L. Thus, the space charge region has a width of only a few Debye lengths. For $v_{f}=$ 10 ms^−1^, corresponding to Peclet numbers Pe_Na_ = 4.49 and Pe_Cl_ = 2.95, respectively, the space charge region is wider, but still in the range of a few nanometers. The filtrate concentration *c*_f_ is around 1.4 mmol/L higher than in the no-convection case. Obviously, the Gibbs-Donnan equilibrium (2) is no longer valid.

**Figure 3.**
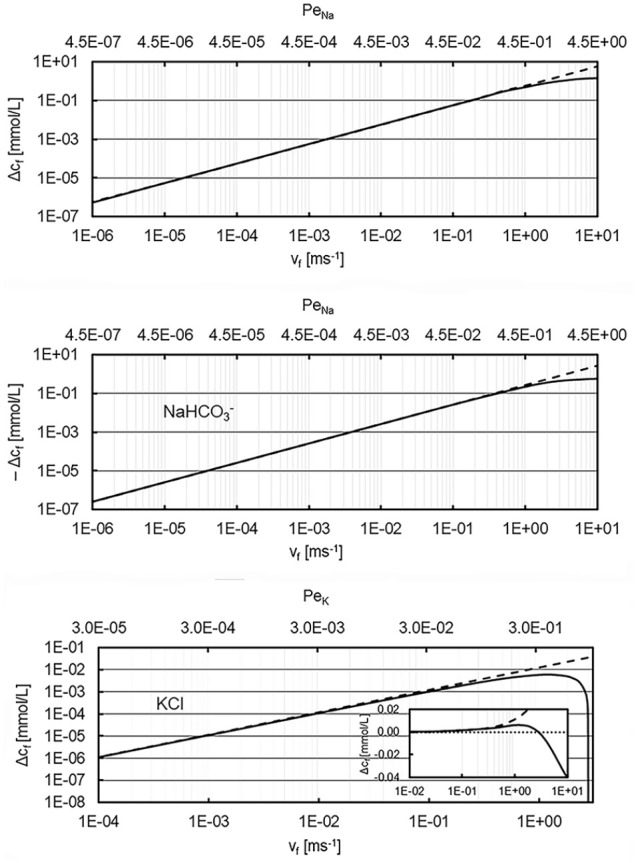
Deviation of filtrate ion concentrations Δcf from the Donnan equilibrium concentration C_eq_ = 154 mmol/L as a function of the transmembrane flow speed for three different solutes (NaCl, NaHCO3, KCl). The upper x-axis shows the corresponding Peclet numbers of the cations. Solid lines: numerical solution, dashed lines: approximative solution (32). Retentate cation concentration 162 mmol/L, Gibbs-Donnan ratio r_d_ = 0.95, temperature 37°C. Note that Δcf for NaHCO3 is negative. In the KCl solution Δcf is negative for vf > 2.7 ms^−1^ so it cannot be displayed on a log-log scale for flow speeds higher than that value. The insert shows Δcf as a linear-log graph for flow speeds up to 10 ms^−1^.

Figure 3 shows the deviation $\Delta c_{f}$ of the filtrate concentration from its zero-convective value for NaCl, KCl, and NaHCO_3_ solutions as a function of the convective flow speed. Again, $c_{f}^{\left(0\right)}$ was set to 154 mmol/L. Similarly, a Gibbs Donnan ratio $r_{d}=0.95$ was assumed. The approximate solution (31) is compared with the numerical solution of equation set (19) over a large range of convective flow speeds.

For flow speeds below 0.1 m/s the approximate solution (32) is an excellent approximation. For higher convective flows, the approximate solution overestimates the deviation from the Gibbs-Donnan equilibrium. The different behavior of the three ionic solutions is due to the different diffusion coefficients of the electrolytes. Since *D*_Na_ < *D*_Cl_, *D*_Na_ > *D*_Bic_, and *D*_K_ ≅ *D*_Cl_, the sign of $\Delta c_{f}$ varies in the three graphs. For NaCl solutions, $\Delta c_{f}$ is always positive, for NaHCO_3_ always negative for $v_{f}$ up to 10 ms^−1^. In the case of potassium chloride, $\Delta c_{f}$ is positive for small flow speeds. In the contrary to the approximate solution, the numerical result reaches a maximum at around 1 ms^−1^, then decreases and crosses zero at around *v* = 2.74 ms^−1^. For higher flow speeds $\Delta c_{f}$ is negative. In this high flow speed region, the complex behavior of the numerical solution is not reproduced by the approximation (32). However, over the full range of flow speeds, $\Delta c_{f}$ for KCl solutions is more than an order of magnitude smaller than that for NaCl and NaHCO_3_. This is due to the fact that for similar diffusion coefficients and a Gibbs-Donnan-ratio close to 1 the two last terms of (32) approximately cancel each other.

**Figure 4.**
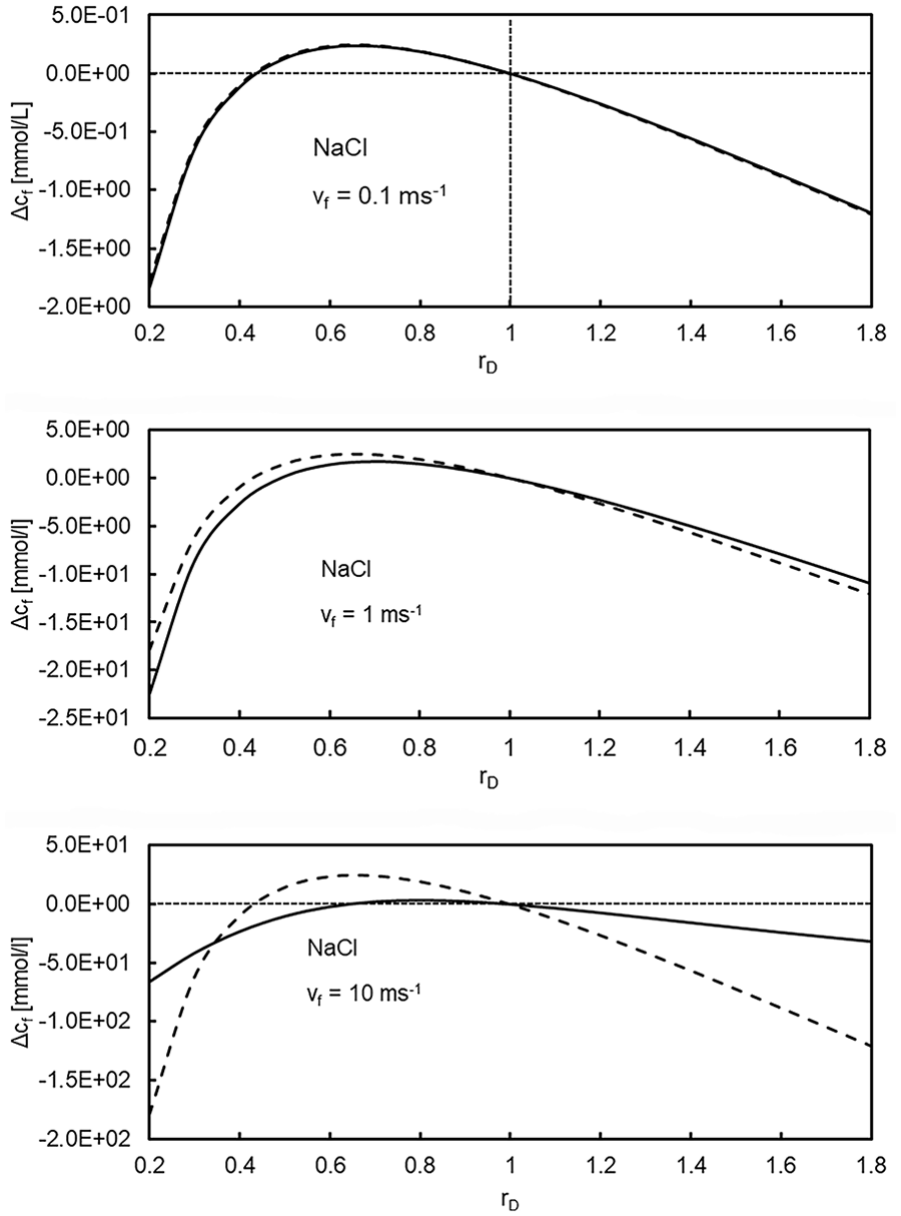
Deviation of filtrate ion concentration ΔC_f_ from the zero-convection equilibrium value C_eq_ = 154 mmol/L as a function of the Gibbs-Donnan ratio r_d_ for convective flow speeds V_f_ = 0.1, 1, and 10 ms^−1^. Solid lines: numerical solution, dashed lines: approximative solution (32).

Figure 4 shows the deviation $\Delta c_{f}$ as a function of the Gibbs-Donnan-ratio *r*_d_ for NaCl at convective flow speeds 0.1, 1, and 10 ms^−1^. As in Figure 3, (32) is a very good approximation for convective flow speeds up to 0.1 ms^−1^. As expected, in the protein-free case *r*_d_ = 1, there is no Gibbs-Donnan effect and consequently the concentrations in the retentate and filtrate are equal. In this case the convective flow has no influence on the ion concentrations.

## Discussion

The Gibbs-Donnan effect induces an ionic concentration difference between the retentate and the filtrate. Note that the retentate concentrations are defined as solute molarities divided by protein-free solvent volume. Inside the membrane pores the permeable ion concentrations approach their filtrate limits within a very thin region. The characteristic width of this region is the Debye length $\lambda_{D}$ . It is several orders of magnitude smaller than the length of the membrane pores, which is around 35 µm for typical hemodialyzer membranes.^17^ In technically relevant filtration processes, the convective flow speed is much smaller than the diffusive speed $\frac{D_{i}}{\lambda_{D}}$ . Using the data in the Results section, one obtains $\frac{D_{Na}}{\lambda_{D}}$ = 2.2 ms^−1^ and $\frac{D_{Cl}}{\lambda_{D}}$ = 3.4 ms^−1^ at a temperature of 37°C. Consequently, the Peclet numbers for permeable ions are usually much smaller than one and the influence of the convective flux on the Gibbs-Donnan equilibrium is negligible. Since the Debye length is inversely proportional to the ion charge *z_i_*, the Peclet numbers for multivalent ions are even smaller than those for monovalent ions.

As an example, from medical practice, in a typical hemofiltration or hemodiafiltration dialysis treatment an ultrafiltrate volume of up to 28 L is obtained in 4 h,^10^ resulting in an convective flow of 117 mL/min. The total area of the dialyzer membrane is typically 1.8 m^2^, however the pores cover only around 20% of this area. This leads to a transmembrane fluid flow speed of 5.4⋅10^−6^ ms^−1^. Assuming all positive ions as Na^+^ and all negative ions as Cl^−^, this results in and. Using equation (32), the resulting deviation of the filtrate concentration is $\Delta c_{f}=6.2\cdot 10^{-6}$ mmol/L, an unmeasurably small value and of no relevance under practical physiological conditions.

Besides its application to hemodialysis, the model may be adapted to similar processes in medicine and industry involving protein separation and purification by semipermeable membranes. Here the protein concentrations may be much higher than in hemodialysis. Formula (32) allows estimating if there is a significant deviation from the Donnan equilibrium or not.

The model has some limitations, however. Firstly, it takes into account only two permeable ion species of opposite charge numbers. A comprehensive treatment would include all ion species. Recently, Waniewski et al.^18^ extended the original Gibbs-Donnan-theory to an arbitrary number of ion species. In this case, equations (13) and (16) could be combined in a similar manner as above and the potential U(x) eliminated. The resulting set of differential equations could be solved numerically.

Alghamdi et al.^19^ and Akbar et al.^20^ describe alternative solution methods for the extended Nernst-Planck equation.

However, since Na^+^ and Cl^−^ are by far the most abundant ions, we do not expect a fundamentally different result by taking into account more than these ion species.

Secondly, the interaction of ions with the membrane pore walls and the mutual ion-ion interaction is neglected. However, in the case of uncharged membrane surfaces the ion-wall electrostatic forces are much weaker than the electrostatic field caused by the volume charge inside the pores. In this case, the ion-wall interaction can be neglected.

In the Nernst-Planck equation (4) the electrostatic potential *U*(*x*) is a mean-field quantity and does not include the charge distribution around individual ions. Consequently, ion-ion interactions are not taken into account. Nevertheless, ion-ion interaction could be included phenomenologically in the Donnan formula (2) by using activities $a_{i}$ instead of concentrations $c_{i}:$

(33) $$a_{i}=\gamma_{i}c_{i}$$

There are several theoretical approaches giving an approximation for the activity coefficients $\gamma_{i}$ . One of the most common is the Debye-Hückel formula^15^ which relates the activity coefficients to the ion strength I:

(34) $$I=\frac{1}{2}\underset{i}{\sum }c_{i}z_{i}^{2}$$

In the case of Gibbs-Donnan ratios $r_{d}$ not too far away from 1, the ion strengths and thus the activity coefficients in retentate and filtrate are nearly equal and approximately cancel out in equation (2). However, the Debye-Hückel theory does not take into account pairing of oppositely charged ions. An alternative theory by Bjerrum and a recent extension of it may give better results on higher ion concentrations.^21^ For the same reasons as above, we do not expect a significant influence on the results.

## Conclusion

The influence of the convective flow on filtrate ion concentration was analyzed using a model based on the extended Nernst-Planck equation. For convective flow speeds below 0.1 m/s an approximative solution allows to estimate the deviation from the Gibbs-Donnan equilibrium. In a typical hemodiafiltration treatment, however, the influence of the convective flow is so small, that no measurable deviation of the filtrate ion concentrations from their Gibbs-Donnan equilibrium values is expected.
